# Linking Precursor Alterations to Nanoscale Structure and Optical Transparency in Polymer Assisted Fast-Rate Dip-Coating of Vanadium Oxide Thin Films

**DOI:** 10.1038/srep11574

**Published:** 2015-06-30

**Authors:** Colm Glynn, Donal Creedon, Hugh Geaney, Eileen Armstrong, Timothy Collins, Michael A. Morris, Colm O’ Dwyer

**Affiliations:** 1 Department of Chemistry, University College Cork, Cork, Ireland; 2 Micro-Nano Systems Centre, Tyndall National Institute, Lee Maltings, Cork, Ireland; 3 Centre for Research on Adaptive Nanostructures and Nanodevices (CRANN), Trinity College Dublin, Dublin, Ireland

## Abstract

Solution processed metal oxide thin films are important for modern optoelectronic devices ranging from thin film transistors to photovoltaics and for functional optical coatings. Solution processed techniques such as dip-coating, allow thin films to be rapidly deposited over a large range of surfaces including curved, flexible or plastic substrates without extensive processing of comparative vapour or physical deposition methods. To increase the effectiveness and versatility of dip-coated thin films, alterations to commonly used precursors can be made that facilitate controlled thin film deposition. The effects of polymer assisted deposition and changes in solvent-alkoxide dilution on the morphology, structure, optoelectronic properties and crystallinity of vanadium pentoxide thin films was studied using a dip-coating method using a substrate withdrawal speed within the fast-rate draining regime. The formation of sub-100 nm thin films could be achieved rapidly from dilute alkoxide based precursor solutions with high optical transmission in the visible, linked to the phase and film structure. The effects of the polymer addition was shown to change the crystallized vanadium pentoxide thin films from a granular surface structure to a polycrystalline structure composed of a high density of smaller in-plane grains, resulting in a uniform surface morphology with lower thickness and roughness.

Metal oxide thin films are an integral part in modern electronic devices and are important in energy storage^1^,^2^, supercapacitors^3^,^4^,^5^, field emission devices^6^,^7^, photovoltaics^8^, thermochromics^9^,^10^,^11^ and as components of the working electrodes in sensing devices^12^,^13^. Improvements in thin film deposition and characterization techniques are of particular interest for high- and low-κ dielectric materials for micro- and nano-electronic devices, where conformal and stoichiometric coatings are required. The deposition methods for producing uniform and stoichiometric thin films in current integrated circuit (IC) devices relies heavily upon methods such as chemical/physical vapour deposition^14^ and sputtering techniques^15^, which can have low sample throughput and high deposition times compared to comparative solution based techniques.

Thin films deposited from solution processed techniques, such as ink-jet printing, spin coating and dip-coating, use a low-cost liquid precursor solution to form morphologically/compositionally uniform thin films^16^. The increased interest in solution processed methods is mirrored by an increase in the development of analytical techniques to correlate precursor and solution changes to their effects on the composition, structure and morphology of the resultant thin film. Better understanding of the factors that affect thin film materials deposited through solution processed techniques is critical for their future uses within modern device infrastructures where the nanoscale characteristics are increasingly important.

As with any other deposition method, many challenges exist and need to be overcome when producing uniform metal oxide thin films through a solution processed technique. Factors affecting thin film depositions from solution processed techniques include deposition technique, additive/dilution effects within the precursor solution, substrate-solution interactions and environmental effects. When choosing the appropriate deposition technique for a solution processed thin film, the desired film morphology and the substrate/device characteristics must be taken into account. As an example, ink-jet printing has been shown to be effective for depositing uniform structures on substrates, however, the printing process can be time consuming and requires a compatible precursor solution which must fit the strict printing systems criteria^17^. The interaction between the substrate and precursor influences the homogeneity of the thin film, where a match can enable epitaxial growth of the desired thin film^18^,^19^. A widely used solution processed technique is dip-coating, which is an alternative to the more costly deposition methods. In dip-coating, through both substrate and precursor selection, a fine control over the thin film thickness and morphology is possible^20^. Dip-coating techniques continue to be improved^21^, and various methods devised for producing thin films with different characteristics include optical constant gradation from a single coating through dip-coating acceleration^22^, and adjustments to the precursor *in-situ* that results in polarity gradients within the thin film^23^. In the cases above, these methods have shown specific benefits for a range of applications, particularly in photovoltaics^24^, where high throughput and cost-efficient dip-coating techniques provide high quality coatings for improved reliability in thin film solar cells.

There are many types of liquid precursors available for use in solution processed techniques, such as sol-gels^25^, organo-metallics, carboxylate and alkoxide-based precursors^16^. As discussed above, the chosen precursor should complement the deposition method and the desired resultant thin film while avoiding undesirable reactivity that might alter the final phase^26^. Polymer assisted deposition (PAD) can be utilized to further influence the characteristics of the thin film through the addition of a polymer to the precursor as an additive^16^,^27^. Avoiding gelation or demixing of constituent inorganic and organic phases during preparation, hydrolysis^28^ and film crystallization^29^ are key requirements for uniform and single phase deposition the thin films. The choice of appropriate precursor and additive is paramount in the formation of metal oxide thin films from solution processed techniques.

For dip-coating techniques, the withdrawal speed influences the formation of the resulting thin films: a low deposition rate produces thin films with a low sample throughput, by increasing the deposition rate, sample throughput is improved but the resultant thin films are often thicker. At dip-coating speeds below ~0.1 mm/s, known as the capillary regime, the formation of the thin film occurs through evaporation at the capillary/air interface and is dependent upon the evaporation of the solvent to form the thin film^22^. Dip-coating speeds at 1 mm/s or above are within the draining regime and at these speeds the formation of the thin film follows the Landau-Levich-Derjaguin model, where gravity-mediated drainage affects the liquid film from which the resultant solid thin film forms after solvent evaporation^30^,^31^.

At deposition speeds within the draining regime, different methods are proposed for increasing the control over the thickness and morphology of the thin films. To prepare thin films with a low thickness and a uniform surface morphology while retaining higher sample throughput using a dip-coating technique, methods must be employed to counter the drawbacks of the draining regime^32^,^33^. One such method, which is used in this work, is to utilise a combination of an alkoxide precursor with the application of a PAD technique that influences viscosity, the nature of the precursor and its conversion to a (uniform) oxide.

Vanadium oxides (VO) are a commonly studied metal oxide material which can be deposited using many different methods including solution processed techniques. Thin films of VO can be produced with a variety of phases, such as vanadium dioxide (VO_2_), vanadium trioxide (V_2_O_3_) and vanadium pentoxide (V_2_O_5_)^34^; the characteristics of each of these phases can vary significantly, and often comprise a mixture of different VO phases^35^. Many phases of VO have applications in areas as intercalation materials^36^, energy storage^37^, electrochromic^38^ and thermochromic devices^39^. Solution processed amorphous and crystalline V_2_O_5_ thin films have recently been demonstrated as hole transport layers in polymer solar cells where uniform defect free surfaces are required^8^,^40^,^41^. By adjusting the crystallisation steps of as-deposited VO from solution processed thin films, it is possible to produce different VO phases in thin film form^9^. Due to its use in a variety of applications, the deposition characteristics of V_2_O_5_ thin films were chosen for study in this work.

For PAD techniques, the choice of polymer additive in the vanadium oxide precursor must a) prevent demixing at any stage during deposition, b) set the viscosity to maintain uniform coating, c) prevent the incorporation of impurities into the film during clean calcination or thermal treatment, and d) provide ordered, continuous or even epitaxial films to the underlying substrate. Challenges for sol–gel processing methods for reactive and multivalent metal-oxides include the identification of a system in which the solvent and organometallic precursors are compatible. Attempts have been made where polymers were added during sol preparation to increase the formation of crack-free critical thin films^42^. However, the formation and understanding of thin films with controllable phase, crystallinity, uniformity and coverage by simple polymer addition remains a significant challenge for solution based depositions of important oxides.

In this work, a combination of techniques was chosen for their compatibility in studying the composition and morphology of solution processed V_2_O_5_ thin films. A low-viscosity alkoxide-based precursor solution, OV(OCH(CH_3_)_2_)_3_, incorporating polyethylene oxide (poly ethylene glycol – PEG) was dip-coated at a fast rate of 2.5 mm/s to form thin films of V_2_O_5_. Microscopic and spectroscopic analysis, including in-plane HRTEM analysis, of the V_2_O_5_ thin films pre- and post-thermal treatment from the PAD method demonstrates the mechanism of thin film formation and conditions required for producing uniform thin films. The influence of PEG-OV(OCH(CH_3_)_2_)_3_ at different alkoxide/IPA dilution ratios prevents thin film demixing that allows pinhole defects to form, minimizes surface roughness and maintains stoichiometric phase and coverage of surfaces from dip-coating, as single films or even multilayers. The film thickness, crystallisation mechanism and optical characteristics (transparency and effective optical bandgap) are influenced by the precursor concentration and degree of crystallization. This work provides a mechanistic understanding for polymer-assisted formation and crystallization of V_2_O_5_ thin films from liquid precursor solutions.

## Results

### Thin Film Characteristics

Alterations to the IPA/alkoxide dilution ratio and the effect of PAD through PEG addition to V_2_O_5_ precursor solutions for thin film solution processed depositions were studied by preparing four precursor solutions and depositing through a dip-coating technique. Each of the solutions was a dilution mixture of V_2_O_5_ alkoxide and IPA with either deionized H_2_O or PEG-400 as an additive. Four types of precursor solution were prepared: a low concentration dilution (LCP) and high concentration dilution (HCP) solution where the additive was deionized H_2_O, the precursor solutions prepared using PEG-400 as an additive were a low (LCP-PEG) and high (HCP-PEG) dilution mixture (see Methods section for more information on precursor solution preparation).

**Figure 1:**
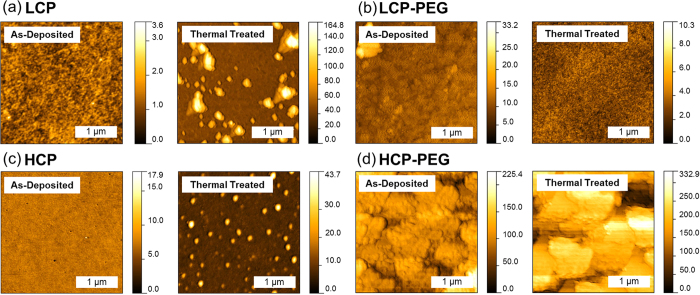
AFM surface images of as-deposited stoichiometric and post-thermally treated orthorhombic V_2_O_5_ films. The films were dip-coated from (**a**) LCP (**b**) LCP-PEG (**c**) HCP and (**d**) HCP-PEG solutions.

AFM surface images of the as-deposited and post-thermally treated dip-coated thin films formed from LCP, LCP-PEG, HCP and HCP-PEG precursors are shown in Fig. 1(a–d) respectively. Corresponding SEM images of the surfaces can be seen in Supplementary Information Fig. S1. The average RMS surface roughness for each thin film obtained from AFM measurements is summarized in Table 1. The as-deposited LCP and HCP thin films have a low RMS roughness value of 0.2 nm and 0.4 nm respectively that increases to 7.8 nm and 4.8 nm after thermal treatment at 300 °C for 14 h. The increase in surface roughness is expected and occurs during crystallization and densification of the thin film coupled with the formation of crystallites on their surfaces during thermal treatment.

The thin films formed from the PEG-containing solutions exhibited a more uniform surface morphology to those without PEG which can be prone to pin-hole development. Factors which affect the formation of pinholes were shown to be both environmental and chemical which can be adjusted through changes to the deposition conditions and through PAD processes^28^. After thermal treatment, the LCP-PEG thin film surface rms roughness decreased from 1.2 nm to 0.2 nm, with a corresponding reduction in the density of observable surface crystallites. The as-deposited HCP-PEG samples had a higher roughness of 8.7 nm which increased to 23.1 nm after thermal treatment. This increase was evident in both the AFM and SEM images of Fig. 1(d) and supplementary Fig. S1 (d) respectively; the surface of the thin films was seen to be covered by large crystals of V_2_O_5_ with evidence of surface cracking and void formation.

**Figure 2:**
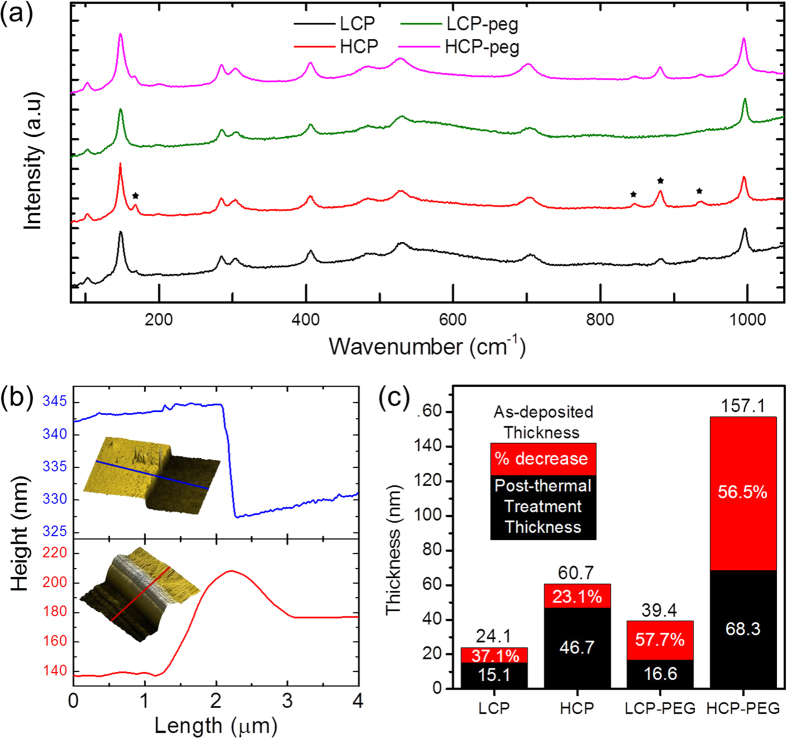
Raman scattering and AFM analysis of dip-coated thin films. (**a**) Raman scattering spectroscopy of orthorhombic V_2_O_5_ thin films formed from different precursor solutions. Vibrations seen at 168 cm^−1^, 845 cm^−1^, 880 cm^−1^ and 935 cm^−1^ are attributed to the formation of small amounts of V_6_O_13_ in the thin films due to a vanadyl-oxygen bond displacement. (**b**) Thickness profiles of a scribed portion of LCP and LCP-PEG thin film with the associated AFM image (inset). (**c**) Thickness of as-deposited and post-thermally treated two layer thin films of V_2_O_5_ formed from each solution. The percent decrease of the thin film due to crystallisation at 300 °C for 14 h is provided.

Raman scattering spectroscopy analysis in Fig. 2(a) confirms the formation of crystalline V_2_O_5_ after thermal treatment for each of the thin films due to the presence of the vibrational frequencies characteristic of orthorhombic V_2_O_5_^43^. Additional vibrations seen at 168 cm^−1^, 845 cm^−1^, 880 cm^−1^ and 935 cm^−1^ are attributed to small amounts of V_6_O_13_ present in the samples due to a displacement of one of the vanadyl-oxygen bonds in the orthorhombic crystal, which can be caused by pseudosymmetry breaking in the layered orthorhombic structure^44^. The vibrational frequencies attributed to these phases is more distinct in thin films formed from the solutions with a high IPA/alkoxide dilution. The peaks at 848 cm^−1^ and 936 cm^−1^ were previously assigned by Su *et al.* to a mode of VO_2_ and the peak at 167 cm^−1^ interpreted as a VO_x_ layered structure^45^. Several reports interpret these symmetric and asymmetric modes as inherent to curvature in the crystal unit cell from VO_x_ nanotubes (VONTs), or a phase seen in VONTs at low annealing temperatures before the nanotube structure is decomposed and re-arranged to form V_2_O_5_^46^,^47^,^48^,^49^. One report does demonstrate the link between these modes and the corresponding XRD pattern, suggesting a mixed phase of V_6_O_13_ and V_2_O_5_^44^.

The V-O-V in-plane mode depicted in Fig. 2(a) can theoretically exhibit an anti-phase stretch with B_2g_ symmetry. This mode is a pseudo centro-symmetric bending in pristine orthorhombic, layered V_n_O_2n−1_. This mode is predicted at ~848 cm^−1^ when the centro-symmetry is broken and the resulting Raman intensity greatly increases. Higher intensity modes at 881 cm^−1^ and 935 cm^−1^ are not predicted based on distortions to the V-O-V and V-O bonds in the D_2h_ space group of orthorhombic V_2_O_5_ and may arise from VO in another phase such as V_6_O_13_ as described above.

To confirm the resultant phase formation in thin films from PAD dip-coating, thicker drop-casted films were also prepared to examine the bulk-like behaviour of the precursor solutions, i.e. to probe if the crystal structure is definitive of the solution composition or influenced by the crystallization mechanism during shear thinning or Marangoni-like instabilities from surface tension variations within the nanofluid precursor solutions (see Supplementary Information, section two). Drop-cast samples allow a comparative bulk-like examination of the final crystal phase and composition. The liquid films hydrolyze to form a solid amorphous thin film of stoichiometric VO which contains the additive, H_2_O or PEG, present in the precursor. The drop-casted samples show the formation of predominantly V_2_O_5_ after thermal treatments identical to that of the thin films. Drop-casted deposits treated at 450 ^°^C showed the formation of tetragonal V_2_O_5_ and monoclinic V_6_O_13_. Regions on the surface of the deposits with varied morphologies were noted which is indicative of the various phases of VO present. Discussion on these phases is presented in the Supplementary Information Figs (S3-S6). Mendialdua *et al.* confirmed through detailed XPS analyses of various VO phases that if carbon contaminated V_2_O_5_ is heated at 300 °C in UHV, it reduces to V_4_O_9_ and subsequently to V_6_O_13_ if further heated to 500 °C^50^.

The thicknesses of the thin films formed from different precursor solutions were studied to examine the effects of concentration and additive changes on the as-deposited and crystallised film thicknesses. Samples were prepared as in a previous study, with two layers of as-deposited VO thin films deposited in a staircase configuration using iterative dip-coating^28^, allowing for the thickness of the single and double layers to be measured using AFM. Figure 2(b) shows two examples of the AFM images and corresponding line profiles for two thin film thickness measurements. The thicknesses of the as-deposited VO first layer on ITO were generally larger then each subsequent layer of VO. The as-deposited first layer thicknesses were 14.3 nm, 39.6 nm, 29.9 nm and 69.3 nm respectively for the LCP, HCP, LCP-PEG and HCP-PEG precursors. An increase in the alkoxide-IPA ratio resulted in an increase in the thickness of each thin film. The HCP film thicknesses were 2.4–2.8 × greater than LCP thin films. An increase in thickness by a factor of 1.6–2.1 × was also found for LCP-PEG thin films compared to those of the LCP films. The HCP-PEG thin films are thickest due to the increase in concentration and the addition of PEG which resulted in a rough, irregular surface. The thickness of single layers of crystallized V_2_O_5_ could not be reliably measured in the LCP and LCP-PEG samples due to the tendency of the thin films to crack during the scribing step; therefore, crystallised thickness effects were studied for double layers of the thin films which could be reliably measured after thermal treatment.

For uniform coverage with good interfacial adhesion to the substrate and on preformed films in iterative or multilayer deposits, the mechanism of crystallization from a nanofluid precursor-polymer mixture is important. The crystallization of the thin films was studied by comparing the as-deposited thickness to the thermally treated thickness of double layer thin films of each solution. Figure 2(c) presents the total thicknesses of two successively dip-coated thin films (as-deposited and thermally treated) from LCP, HCP, LCP-PEG and HCP-PEG precursor solutions.

### Optical Transmission of Uniform Dip-coated Films

**Figure 3:**
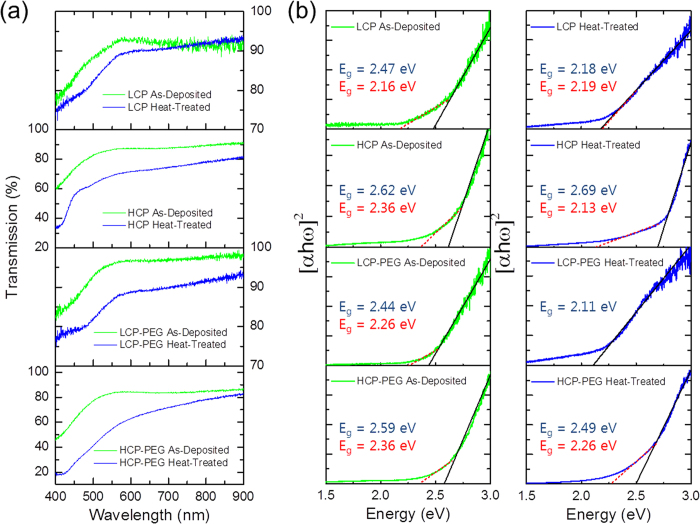
Optical transmission of VO thin films. (**a**) UV-Vis transmission spectroscopy of as-deposited and post-thermally treated V_2_O_5_ thin films formed from both low and high dilution solutions with/without PEG. (**b**) Plot of [αhω]^2^ versus photon energy for the as-deposited and thermal-treated thin films. For the thin films which exhibit the secondary energy gap, the values are shown in red.

The optical transmission of the polymer-free and PAD amorphous and crystallized thin films is shown in Fig. 3(a). All films, at low or high solution dilutions and with or without polymer addition, show a decrease in optical transmission after thermal treatment and crystallization. Thin film densification and additive removal occurs during the thermal treatment and crystallization step. Figure 3(a) shows that the as-deposited LCP-PEG thin films have a higher transmission (>80-95%) in the visible region beyond their respective absorption edges, in spite of being 1.6–2.1 × thicker than LCP thin films. Uniform mixing of the PEG-blended solution decreases the opacity of the thin films prior to crystallisation, and based on microscopy analyses (*vide infra*), these films are inherently more uniform, preventing scattering at wavelengths greater than the absorption edge. These thicker films are largely composed of the PEG-400 polymer with amorphous V_2_O_5_ inclusions and thus are more transparent than thin films that are composed of the amorphous V_2_O_5_ inclusions alone. Drude absorption beyond the plasma frequency is not a contributory factor in the visible frequency range for these films, as the thickness is sub-wavelength (less than *λ*/30 at 650 nm for LCP and LCP-PEG).

As the effect of substrate interface reflections is taken into account by referencing (subtracting) the transmission of the substrate alone from that of the coated substrate, the differences in transmission are thus dominated by the reflectivity caused by crystallization (reduction in void content and increase in refractive index) and by PEG removal in polymer-assisted deposited thin films. Frequency-dependent thin film interference oscillations are not found in the visible range while the drop in transmission is characteristic of these compact crystalline thin films.

The effective optical bandgap (*E*_g_) of the VO thin films after thermal treatment was calculated by employing the modified Davis-Mott relation in Fig. 3 (b) and presented as Tauc plots^51^. The absorption coefficient is related to the band-gap through the relation 
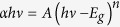
 where *α* is the absorption coefficient of the thin films, *A* is a constant and *E*_g_ is the effective optical bandgap. The exponent *n* is related to the type of transition for the material, in this case *n* = 2 for an indirect allowed transition, determined where [*αhω*]^2^ = 0. The index step change from glass to ITO has been accommodated in the effective gap fits in Fig. 3.

A lower indirect optical transition energy is found in PEG-containing thin films: the effective bandgap values for the LCP and HCP thin films are 2.18 eV and 2.69 eV, while those for the LCP-PEG and HCP-PEG films are 2.11 eV and 2.49 eV respectively. An increase in the dilution of solutions for LCP to HCP and LCP-PEG to HCP-PEG thin films is correlated with a blue-shift in the effective optical bandgap by 0.51 eV and 0.38 eV, respectively.

Scattering from nano-inclusions or from discontinuities in any of the films is discounted as the primary reason for the shift in absorption, due to sub-wavelength sizes and Rayleigh scattering from solidified features in a solid film 20–100 nm in thickness is less likely to influence the optical transmission. LCP and LCP-PEG films on ITO glass comprise two interfaces with their individual refractive index contrast. In order to explain contributory reasons to reduction in transmission intensity after crystallization and densification in uniform films pre- and post-heating, we consider reflection losses during transmission at two interfaces, where the film is as-deposited, mixed with PEG (and thus thicker), or crystallized and more dense. Thus from wave optics, the reflectivity is related to the modulus of the electric fields via the three-phase Fresnel equations for light reflection at the ITO-VO interface (*i*) and that at the VO thin film-air interface (*j*), and can be expressed as 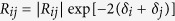, where 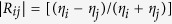 and the phase thickness of each layer in the film-substrate system is 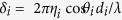, where 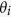 is the angle of refraction and *d*_*i*_ is the actual layer thickness. As with antireflection coatings for instance, the changes in refractive index of each film reported here (at thicknesses much less than *λ*/4) should affect the total transmittance, and measured values are thus related to thickness and effective index based on phase and structure. The reflectance from each interface of the two-film system for each type of thin film prepared that reduces total transmittance, 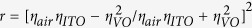 are summarised in Table 1. Using the Moss relation, the effective bandgaps from Fig. 3 were related to the refractive index according to 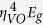 ~ 95 eV, and the values are shown in Table 1. Since the ITO transmittance and reflectance are subtracted as background in Fig. 3 (a), the variations in transmission at 650 nm, are influenced by effective index contrast reflectance at the VO-air interface, as the reduction in thickness of the film (with and without PEG and pre- and post-heating) does not account for the decrease in optical transmission at wavelengths where band edge material absorption does not occur.

### In-Plane HRTEM of PAD Processes in Oxide Thin Films

**Figure 4:**
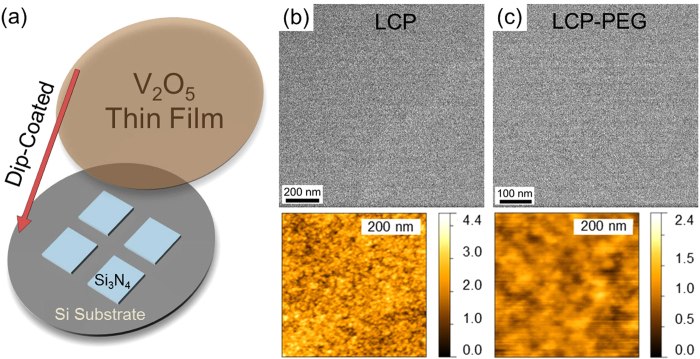
AFM and electron microscopy of dip-coated thin films. (**a**) Schematic of Si_3_N_4_ membrane TEM grids with dip-coated V_2_O_5_ thin films for investigation of their in-plane structure. In-plane TEM images and corresponding AFM images of as-deposited stoichiometric V_2_O_5_ thin films dip-coated from (**b**) LCP and (**c**) LCP-PEG solutions.

Thin films were dip-coated from the LCP and LCP-PEG precursor solutions onto the surface of the Si_3_N_4_ TEM grids, allowing examination of in-plane crystal structure by HRTEM, high resolution AFM and Raman scattering spectroscopy. A schematic of V_2_O_5_ dip-coated onto a Si_3_N_4_ coated TEM grid is shown in Fig. 4(a): the in-plane structure of the films can be probed by HRTEM without complications from separate entities of material overlapping in projection as found in TEM samples prepared through exfoliation methods. Single thin film layers deposited from the low dilution concentration solutions were formed on the Si_3_N_4_ TEM grids so that the effect of PEG on the in-plane crystallization could be examined. HRTEM and high resolution AFM (inset) images for the LCP and LCP-PEG as-deposited thin films are shown in Fig. 4(b,c) respectively. There is no evidence of crystalline structure evident prior to thermal treatment in the HRTEM images for either deposit as expected from the XRD and Raman scattering spectroscopy performed previously in this work. High-resolution AFM images show that the amorphous LCP and LCP-PEG films have a granular surface morphology where the individual features are composed of inclusions of stoichiometric amorphous V_2_O_5_ formed during a nanofluid step as outlined previously^28^. The LCP-PEG samples appear to have comparatively larger individual inclusions. These larger inclusions are attributed to the continual coarsening of hydrolyzing alkoxide into solid V_2_O_5_ and the development of instabilities in the PEG that are retarded by the increasing density of inclusions with time.

**Figure 5:**
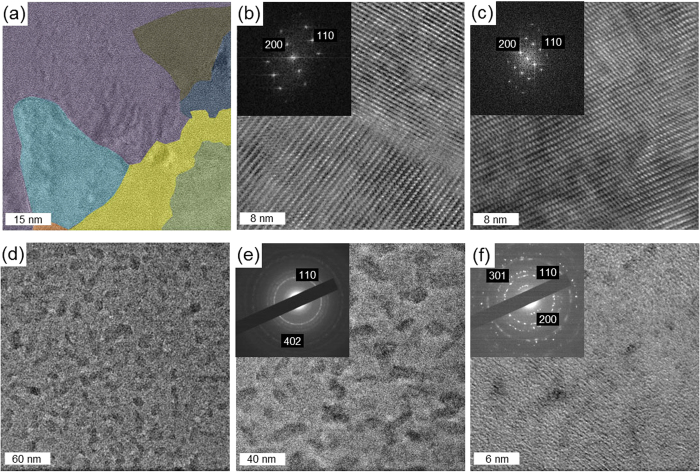
Post-thermal treated in-plane HRTEM. Images and corresponding FFT or diffraction patterns of orthorhombic V_2_O_5_ thin films formed from (**a–c**) LCP and (**d–f**) LCP-PEG solutions. Thin films formed from LCP precursor solutions show the formation of grains of orthorhombic V_2_O_5_ with defined grain boundaries. Thin films formed from LCP-PEG precursors have a polycrystalline orthorhombic V_2_O_5_ structure.

The V_2_O_5_ coated grids were thermally treated for 14 hours at 300 ^°^C and the HRTEM and AFM analyses were repeated. Figure 5(a–c) shows the HRTEM and related fast Fourier transforms (FFT) patterns for the thermally treated LCP samples, while Fig. 5(d–f) shows the corresponding images for the LCP-PEG thin films. The LCP thin film crystallization results in individual crystalline grains of orthorhombic V_2_O_5_ in a patchwork arrangement; the different grains indexed to orthorhombic V_2_O_5_ are highlighted in Fig. 5(a–c) and in Supplementary Information Fig. S7.

The LCP-PEG thin film shown in Fig. 5(d) has a pockmarked morphology in the HRTEM images where the presence of individual crystalline grains is not apparent as found with the LCP thin films. In such films, the removal of the polymer results in high density, small grains that form a polycrystalline film (Fig. 5(e,f)), which from Fig. 1, is uniform and of low surface roughness. Polycrystalline orthorhombic V_2_O_5_ is also found at higher magnification (Fig. 5(f)), and single crystalline grains are not characteristic of calcined PAD V_2_O_5_ thin films at atomic resolution. The data confirm that polymer-assisted synthesis using PEG-containing precursors alters the hydrolysis and gelation point of the nanofluid, forming high density grains within the structure. Regions of the thermally treated LCP-PEG thin films exhibited a porous morphology as seen in Supplementary Information Fig. S8. These regions are small and interspersed on the sample and show an increase in the porosity of the thin films due to the effects of the PEG on the hydrolysis and gelation point of the nanofluid stage.

**Figure 6:**
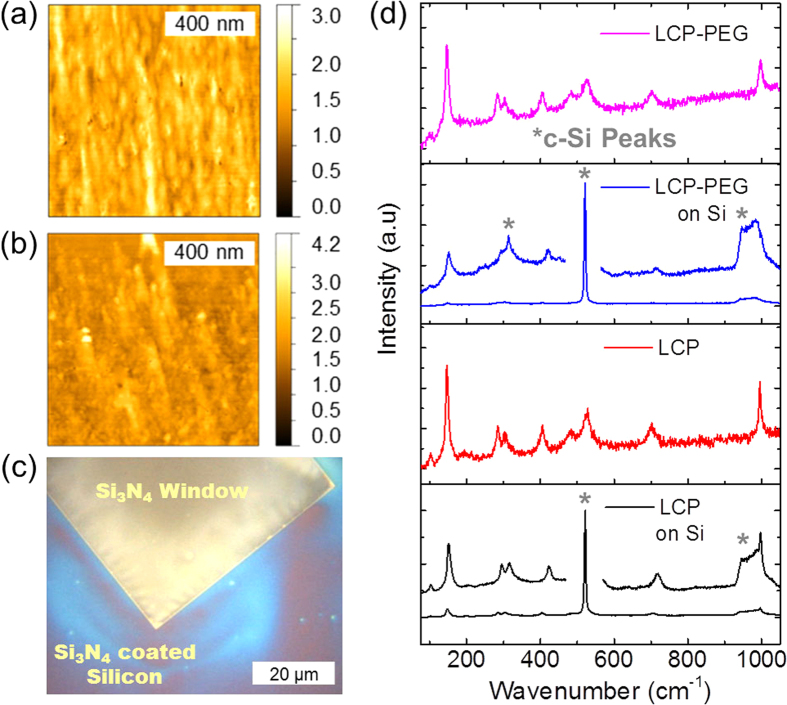
AFM surface images. Analysis of (**a**) LCP and (**b**) LCP-PEG orthorhombic V_2_O_5_ thin films deposited onto Si_3_N_4_ TEM grids after thermal treatment. (**c**) Optical microscope image of a thin film of V_2_O_5_ on a Si_3_N_4_ coated TEM grid. (**d**) Raman scattering spectroscopy of the thin films formed on Si_3_N_4_ windows. The vibrational contributions from the underlying Si substrate are highlighted by asterisks (*).

High-resolution AFM images of the thermally-treated LCP and LCP-PEG thin films on the Si_3_N_4_ TEM grids are shown in Fig. 6(a,b) respectively. The single crystalline grains of V_2_O_5_ for the LCP thin films are seen in Fig. 6(a) as found in the corresponding HRTEM images. The pockmarked and smaller polycrystalline structure of the LCP-PEG thin films can be characterized from Fig. 6(b) due to the presence of smaller structures and a similar surface morphology as seen in the HRTEM images.

Raman scattering spectroscopy was performed on the V_2_O_5_ thin films deposited on the Si_3_N_4_ coated TEM grids after thermal treatment. Figure 6 (c) is an optical microscopy image of the surface of the grids showing the thin films deposited onto the Si_3_N_4_ window and the surrounding regions. The Raman scattering spectra shown in Fig. 6(d) confirm the formation of orthorhombic V_2_O_5_ for both the LCP and LCP-PEG thin films after thermal treatment. The low intensity of the vibrational modes is attributed to the thinness of a single dip-coated film on the Si_3_N_4_.

## Discussion

As described by Sanchez *et al.* the role of chemical modifications to alkoxide based liquid solutions is to affect the hydrolyzation and condensation reactions such that the resultant solution is adjusted and improvements to the deposition of the solid materials can be made^52^. Both the H_2_O and PEG-400 additives can have different effects on the formation reaction of the alkoxide based precursor due to their specific chemistry. The low volume of the H_2_O additive is insufficient to fully hydrolyse the precursor solution. The addition of the H_2_O triggers the hydrolysis of the liquid thin film in the initial stages of the deposition stage, so that the solid thin film better adheres to the surface of the substrate during its formation. The source of H_2_O reactant for the hydrolysis of the deposited liquid film for forming a solid thin film comes from the deposition environment after the initial dip-coating. The role of PEG-400 as an additive is to form a mixed vanadium-PEG alkoxide derivative in the liquid solution; during the formation reaction with environmental H_2_O the effects of the polymer on the hydrolyzation and condensation alters the morphology of the resultant thin film^52^. The effect of the PEG additive influences mixing and demixing within the as-deposited thin film before and during thermal treatment. The different alterations to the precursor solution affect the structural, morphological and optoelectronic properties of the thin films in a variety of ways as shown in the results above.

The roughened surface morphology seen in the HCP-PEG thin films is attributed to the higher concentration of PEG and alkoxide within the as-deposited thin film. During the thermal treatment, the PEG demixes and carbonizes, while the amorphous VO crystallizes into orthorhombic V_2_O_5_. The as-deposited AFM images in Fig. 1 for the HCP-PEG films show an already roughened surface where the PEG has non-uniformly spread through the thin film during the deposition process. As the temperature further increases, the demixed PEG is removed from the film and results in surface fracturing and the formation of nanoscale sub-wavelength voids in the solidifying V_2_O_5_. Uniform films before and after thermal treatment are possible from LCP-PEG thin films where an optimum dilution between the IPA, alkoxide and PEG allows for decomposition and removal of the PEG additive while the crystallizing V_2_O_5_ forms a coherent thinner thin film (Fig. 1(b)).

The relative thickness reduction for PEG-containing solutions is consistently highest after heating at 300 ^°^C for 14 hours. Using the more diluted concentration of alkoxide increases the rate of hydrolyzation compared to LCP-PEG thin films where the PEG spreads uniformly through the film. Consequently, AFM and thickness measurements indicate that discontinuous demixing and subsequent PEG decomposition affects the overall uniformity. Crystallized film uniformity is improved through a PAD technique with the addition of PEG, but with lower IPA/alkoxide concentration.

The processes which control the formation of alkoxide based thin films dip-coated within the draining regime were examined. Dynamic effects such as nanocluster formation of V_2_O_5_ during hydrolysis, which prevents dewetting, and associated changes in overall film composition during the alkoxide decomposition are well documented for polymer and liquid films containing nanoparticles and nanostructures, the details of this work are presented elsewhere^28^. At dip-coating rates within the Landau-Levich-Derjaguin draining regime, Faustini *et al.* reaffirm that thin film thicknesses can be constrained, with minimal variation, for relatively fast deposition rates of ~1–10 mm/s^22^,^31^.

The thickness of a liquid coating that remains on the surface of a substrate at high speed withdrawal rates is dependent upon gravity induced drainage which dominates over capillary and meniscus effects (See Supplementary Information Fig. S9 for differences between fast- and slow-rate dip-coated depositions). Therefore, the thickness of the liquid coating is related to the drainage balancing surface tension such that 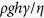 ~ u where *u* is the withdrawal rate, *h* is the liquid thin film thickness, *γ* is the surface tension, *η* is the viscosity, *ρ* is the solution density and *g* is the standard gravity. At faster deposition rates that initially overcome gravitational forces (>1 mm/s), the thickness of the liquid film can be approximated with 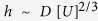, where *D* can be calculated using constant values of the deposited liquid, 
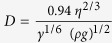
22,30.

For each precursor solution studied in this work, the low molar volume fraction of the alkoxide and additive at both concentrations approximates to that of the density of the main component, IPA. The impact of the alkoxide and additive on the wetting characteristics of the IPA is also negligible due to their small comparative overall volume within the solution. Therefore, the thickness of the IPA film at a withdrawal speed of 2.5 mm/s can be approximated by using the constants for IPA. Using literature values for IPA (γ = 2.212 × 10^−3^ kg/m, ρ = 786 kg/m^3^, η = 2.3703 × 10^−3^ kg/ms) an estimate for the static liquid film thickness prior to drainage and formation of the solid thin film through hydrolysis formed at high withdrawal rate of 2.5 mm/s is 9–10 μm.

To account for the transition from a liquid to solid thin film through evaporation processes, a material proportion constant (*k*) term can be introduced such that h ~ 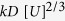. The *k* term is specific to the initial precursor in each case and is a volume proportion constant for the material mixture within the liquid solution and defined by 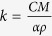 where *C* is the inorganic precursor concentration, *M* is the inorganic material molar weight, *α* is the fraction of the material in the film and *ρ* is the density of the inorganic material^30^. An estimate for an equivalent *k* value for the LCP and HCP precursors is 0.7636 and 0.7962 respectively. A *k* value can be calculated for each solution using the experimentally obtained thin film thickness; the calculated *k* for the LCP and HCP precursors is 1.6858 × 10^−3^ and 4.0756 × 10^−3^ respectively, which are two orders of magnitude lower than that of the estimates. (The calculation of both *k* and *h* is further discussed in Section four of the Supplementary Information with the calculated and estimated *k* values for each of the precursors provided in Supplementary Information Table S1).

Typically, *k* is cited for sol-gel systems with a large volume fraction of inorganic material. In the present case, the molar volume fraction of inorganic V-O species in each solution is several orders of magnitude smaller than that of the IPA. Also, unlike many sol-gel systems, thin films formed from alkoxides and carboxylates do not form through evaporation of the solvent alone, but also convert from one compound to another when transitioning from their liquid film phase to a solid thin film phase due to hydrolysis and condensation effects. To explain the difference in the estimated and calculated *k* values the formation processes of the thin film were examined optically during deposition.

**Figure 7:**
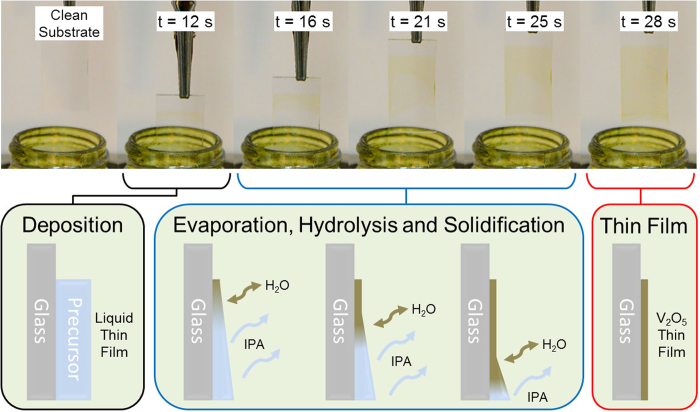
Optical images of a glass substrate being dip-coated over time. The formation process from a liquid solution film to a solid stoichiometric V_2_O_5_ thin film through a combination of evaporation, hydrolysis and solidification is shown optically and schematically.

The formation process of the V_2_O_5_ thin films during deposition is demonstrated in Fig. 7, where the initial liquid film is first deposited onto the substrate using fast-rate dip-coating to an estimated thickness of ~9.7 μm. The solid V_2_O_5_ thin film forms through the evaporation of IPA and the subsequent hydrolysis of the alkoxide and additive into a solid thin film; a process which begins at the top of the deposit and proceeds down the substrate over time. This formation process is shown in the optical images of Fig. 7 and the accompanying movie in the Supplementary Information.

The change in thin film thickness occurs during the short hydrolyzation and condensation step after IPA evaporation. The liquid/solid phase change of the alkoxide precursor is the mechanism through which the solid thin films are formed after the initial liquid film is formed; the estimated *k* value does not take this change into account resulting in a lower estimated thin film thickness. The experimental results provide an empirical value for *k*, which takes into account the phase change processes for each of the precursors. It is due to this phase change process that thin films with nanometre scale thicknesses can be deposited at a dip-coating rate of 2.5 mm/s within the Landau-Levich draining regime.

The effects of the PEG additive within the thin film pre- and post-thermal treatment is shown to influence the long range order of the deposit and therefore the optical characteristics of the thin film are affected. The higher dilution concentration of alkoxide/PEG to IPA in the HCP-PEG thin films and the influence of PEG addition respectively results in a different morphology, surface roughness, thickness (see Figs 1 and 2) and optical transmission characteristic for the HCP and LCP-PEG thin films. The profile of the transmission spectra for the HCP-PEG thin films in Fig. 3(a) shows a discontinuity when compared to the spectra for the corresponding HCP thin film, particularly after thermal treatment. During deposition, the PEG does not have sufficient time during the hydrolyzation step to spread homogenously throughout the thin film; instead the PEG clusters around the amorphous V_2_O_5_ inclusions resulting in a rougher surface morphology as shown in the AFM data above. The rougher morphology subsequently allowed for selectively higher PEG decomposition during the thermal treatment resulting in the formation of voids and surface fracturing as was also observed for the drop-cast films shown in supplementary Fig. S3 (b).

This change is attributed to an increase in disorder within the thin films from the higher IPA:alkoxide dilution which resulted in a more densely packed thin film with a higher optical conductivity. The thin films crystallise from amorphous deposits, but optical data is acquired at room temperature, thus no insulating phase transition accounts for a change in optical conductivity and transparency. The presence of other phases of reduced vanadia may explain absorption due to intra-bandgap states; the exact reason is as-yet unclear. The films formed from these precursors can comprise some V_6_O_13_ and tetragonal V_2_O_5_ as described in detail in Supplementary Information section two, and supplementary Fig. S4 and Fig. S5, altering the total absorbance of the thin films.

Oxygen emission at temperatures >300 °C is known to occur in V_2_O_5_, and in the present case of thin films from PAD methods, other phases such as tetragonal V_2_O_5_ and monoclinic V_6_O_13_ result from vanadyl oxygen vacancy formation in orthorhombic V_2_O_5_. Creating donors due to oxygen vacancies can increase optical conductivity (*n* = [donors] – [acceptors]), and lower transparency (or at least shift the onset of absorption due to a Burnstein-Moss shift). Based on HRTEM analysis of the crystallization mechanism and the densification of nanoscale crystallites of V_2_O_5_ during thin film formation, the reduction in optical transparency is likely caused by changes to the optical constants by an increase in the effective refractive index. Our measurements confirm that inherent porosity and voids of non-V_2_O_5_ regions may define an effective medium with a lower effective index^53^,^54^,^55^. These thin film optical coatings with uniform coverage provide higher transmission without undergoing a metal-insulator transition (MIT) due to a phase change, but alter optical constants due to the nanofluid-like composite structure of the coating prior to crystallization and densification. In trying to explain MIT effects in V_2_O_5_ thin films^56^ the structure of the film and void content, which can significantly alter optical constants and provide high grain-density resistance paths for conduction should be considered, as it defines the optical conductivity and transparency of conductive thin films^57^.

While the amorphous as-deposited films (with and without PEG) have optical transitions indicated by extrapolation from a single linear region in Fig. 3(b), the crystallized thin films show two linear regions at energies higher than the Urbach tail, suggesting absorption contributions from multiple VO phases. For the thin films that exhibit this lower energy gap the absorption edge and value are highlighted in red in Fig. 3(b). The energy difference between the higher and lower energy region in the crystallized thin films is larger for the films from high-dilution precursors.

The thermal processes that lead to different thickness, uniformity, roughness and crystallinity of the V_2_O_5_ thin films after thermal treatment were further studied by dip-coating onto Si_3_N_4_ TEM window grids. Analogous to liquefied polymer films, dilution which alters the molecular weight of the overall precursor also alters the viscosity, density of V_2_O_5_ formation and thus processes and forces associated with dewetting and phase separation^28^,^58^,^59^. The dynamic formation of V_2_O_5_ inclusions during hydrolysis is similar to nanoparticle covered and nanoparticle-embedded polymer films, where the nanoparticles prevent dewetting and control the development of patterning and roughness development during solidification^60^,^61^,^62^. Embedded nanoscale inclusions can potentially enhance the stability of films formed during polymer-assisted deposition, yet the process of oxide formation during film growth under faster withdrawal speeds that negate dominant effects of meniscus and drying fronts at the air-liquid interface (hydrolysis occurs *after* the dip-coating in the ‘draining’ regime, which defines the thickness) has not been investigated.

In-plane HRTEM, AFM and Raman scattering spectroscopy showed that the presence of PEG in the as-deposited thin films of VO does not impede the formation of orthorhombic V_2_O_5_ after a thermal treatment at 300 ^°^C. The effect of the removal of PEG from the LCP-PEG samples was only evident in the formation of a pockmarked structure which was visible by HRTEM imaging shown in the Supplementary Information Fig. S8. The comparison between the surface morphology of thin films produced from a low dilution solution with/without PEG addition shows that the surface morphology of these thin films differs in their smoothness as shown by AFM. Thin films formed without the addition of PEG have a surface composed of single crystalline grains of V_2_O_5_ with well-defined in-plane grain boundaries on the atomic scale. Thin films prepared with the addition of PEG are composed of a polycrystalline V_2_O_5_ due to the heterogeneously mixed polymer in the as-deposited thin films necessitating the formation of small crystal grains.

## Conclusions

Thin films of V_2_O_5_ of varied surface morphologies and characteristics were deposited using fast-rate dip-coating such that the effects of PAD and dilution changes between the alkoxide/additive pair and the IPA solvent of an alkoxide based precursor were studied. Alterations to the precursor solution were shown to have significant effect on the thin film morphology and structure both pre- and post-thermal treatment. The optical properties, including conductivity and transparency associated with non-uniform demixing of polymers within the thin films, was also examined for the resultant amorphous as-deposited thin films and shows that uniform mixing of the polymer additive within the amorphous film is required to maintain long range order. The production of thin films by single dip or iterative dip coating at high rate with sub 100 nm thicknesses is possible due to the phase change of the alkoxide based precursor during hydrolysis and condensation after the formation and subsequent evaporation of the carrier solvent film.

Thin films containing discontinuities that contribute to higher roughness via demixing/dewetting effects, contain surface cracks and surface-bound crystallites when crystallized. The presence of other phases of VO was also found for these samples and was attributed to pseudosymmetry breaking in the layered orthorhombic crystal structure: the small proportion of extra phases of VO are likely contributors to additional absorption in the transmission spectra as the thickness and discontinuities are sub-wavelength and do not contribute to significant scattering. Thin films deposited from a low dilution precursor solution with the addition of PEG-400 for PAD effects produced the most uniform thin films of orthorhombic V_2_O_5_ with a low surface roughness both pre- and post-crystallisation. In these thin films, a significant presence of other VO phases was not found through either Raman scattering or UV-Vis spectroscopy.

Using a combination of HRTEM, AFM and Raman scattering techniques, the effects of PAD on the crystallisation of V_2_O_5_ thin films were studied in an in-plane configuration. PAD effects on the V_2_O_5_ thin films were shown to alter the crystallisation mechanism such that a polycrystalline structure composed of small high density grains was formed as opposed to a larger granular structure which forms without PAD effects. The smaller grains of the thin films formed through PAD processes are attributed to the heterogeneous mixing of PEG within the as-deposited thin film resulting in smaller crystal grains forming during thermal treatment. The small crystal grains were shown through in-plane HRTEM and AFM to result in a uniformly smooth surface morphology with a low rms roughness.

The study of the formation characteristics and subsequent analysis of alkoxide based fast-rate dip-coated thin films in this work indicates a promising method for optoelectronic oxide material deposition in thin film transistors, functional coatings and as oxides for electronic devices that are processed on flexible substrates or plastic electronics (amorphous room-temperature oxides, or crystallized films on polyimides for example). The applications of the PAD process is potentially a useful solution-processable low cost approach for controlling the types of thin films that can be deposited. By cataloguing the effects of the precursor alterations, unwanted thin film attributes such as roughness can be systematically removed, or enhanced for influencing light-matter interactions as desired. This particular approach does not necessitate new precursor development and outlines how the rheology and chemical conversion characteristics of precursor solutions can provide a range of uniform thin films.

## Methods

### Precursor Synthesis

Four types of precursor solutions were prepared to produce V_2_O_5_ thin films where the dilution of the alkoxide to the solvent and also the effect of two different types of additives to the precursor could be studied. Vanadium (V) oxytriisopropoxide OV(OCH(CH_3_)_2_)_3_ and PEG-400 was purchased from Sigma-Aldrich, and isopropyl alcohol (IPA) was used as-received.

For preparing the low and high dilution precursors, the IPA was mixed by volume with the vanadium oxytriisopropoxide precursor and the chosen additive at a volume ratio of 1000:10:1 and 250:10:1 (IPA : Alkoxide : Additive) respectively. Deionized water was added as an additive to the precursor solution to aid the hydrolysis and was replaced by the same liquid volume of PEG-400 in the polymer-assisted deposition (PAD) precursor solutions. The prepared solutions were stored in closed vials with 4 Å molecular sieves from Sigma-Aldrich to increase their shelf life by preventing hydrolysis from the environment prior to the deposition. As mentioned in the main text, four types of precursor solution were prepared: a low concentration dilution (LCP) and high concentration dilution (HCP) solution where the additive was deionized H_2_O, the precursor solutions prepared using PEG-400 as an additive were a low (LCP-PEG) and high (HCP-PEG) dilution mixture.

### **V**_**2**_ **O**_**5**_ Thin Film Preparation

A drop-casting technique was used to prepare bulk-like samples of the materials while dip-coating was performed using a PTL-MM01 desktop dip-coater at a constant withdrawal rate of 2.5 mm/s for depositing uniform thin films.

Samples were prepared on high quality ITO-coated glass substrates purchased from Sigma-Aldrich with a nominal ITO thickness of 15–30 nm. The substrates were cut to be 12.5 mm in length and 5 mm in width. The crystallization step was performed in a convection oven ramped up to 300 ^°^C at a rate of 1 ^°^C/min and held for 14 hours. For higher temperature thermal treatments, a tube furnace was used at a maximum temperature of 450 ^°^C for 14 hours at a ramp rate of 1 ^°^C/min.

### Characterization Techniques

Surface morphologies were examined using scanning electron microscopy (SEM) and atomic force microscopy (AFM). SEM analysis was performed on an FEI Quanta 650 FEG high resolution SEM equipped with an Oxford Instruments X-MAX 20 large area Si diffused EDX detector. Images were collected at an operating voltage of 10–20 kV. AFM was used to analyse the surface topography and roughness of the prepared samples. Scans were performed on a Park XE-100 AFM system in non-contact mode with SSS-NCHR enhanced resolution tips, the XY and Z resolution are ~2 nm and 0.05 nm respectively. Samples were scribed with a scalpel blade and AFM images were taken at the interface between the substrate and thin film surface to determine the thin film thickness.

To determine the phase of the films post-crystallisation, Raman scattering spectroscopy was collected with a Renishaw InVia Raman spectrometer using a 514 nm 30 mW Argon Ion laser, spectra were collected using a RenCam CCD camera. The beam was focused onto the samples using either a 20 ×/50 × objective lens. X-Ray Diffraction (XRD) analysis was performed using a Phillips Xpert PW3719 diffractometer using Cu Kα radiation (40 kV and 40 mA) scanned between 10^°^–40^°^.

UV-Vis transmission spectroscopy was performed by illuminating samples with white light from a Halogen bulb incident normal to the sample surface and collimated to a beam diameter of ~1 mm. Spectra of the transmitted light were collected using an Ocean Optics USB2000+VIS-NIR-ES CCD spectrometer in the wavelength range 200–950 nm. A baseline of the corresponding ITO substrate was taken before each scan.

The in-plane structure of the thin films was determined through HRTEM using a JEOL 2100 high resolution TEM at 200 kV. Single layer thin films were dip-coated onto the surface of 15 nm coated Si_3_N_4_ TEM grid windows. Thermal treatments were performed in a conventional oven at 300 °C in air for 14 hours.

## Additional Information

**How to cite this article**: Glynn, C. *et al.* Linking Precursor Alterations to Nanoscale Structure and Optical Transparency in Polymer Assisted Fast-Rate Dip-Coating of Vanadium Oxide Thin Films. *Sci. Rep.* **5**, 11574; doi: 10.1038/srep11574 (2015).

## Supplementary Material

Supplementary Information

Supplementary Information

## Figures and Tables

**Table 1:** Precursor solution and thin film data for orthorhombic V _2_O_5_ thin film samples prepared in this work.

Precursor Solutions	Avg. Thin Film RMS Roughness (nm)		Avg. Second Layer Thickness (nm)		Calc. E_g_ (eV)		Effective 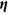		Interfacial reflectance, *r*	
	As-deposited	Thermal Treated	As-deposited	Thermal Treated	As-deposited	Thermal Treated	As-deposited	Thermal Treated	As-deposited	Thermal Treated
LCP	0.2	7.8	24.1	15.1	2.47	2.18	2.49	2.57	0.29	0.31
LCP-PEG	1.2	0.2	39.4	16.7	2.44	2.11	2.50	2.59	0.29	0.32
HCP	0.4	4.8	60.7	46.7	2.62	2.69	2.45	2.44	0.28	0.27
HCP-PEG	8.7	23.1	157.1	68.3	2.59	2.49	2.46	2.48	0.28	0.29

The refractive index of ITO is 1.848 at 650 nm. *r* values calculated at 650 nm. Note, the transmission values in Fig. 3 have the ITO interface background subtracted.
