# Supplementary material for: Linking Precursor Alterations to Nanoscale Structure and Optical Transparency in Polymer Assisted Fast-Rate Dip-Coating of Vanadium Oxide Thin Films
Source: Sci Rep. 2015 Jun 30;5:11574. doi: 10.1038/srep11574 (PMC4485054; doi:10.1038/srep11574)
Supplement: Supplementary Information [file srep11574-s1.pdf]

Linking Precursor Alterations to Nanoscale Structure and Optical Transparency in Polymer Assisted Fast-Rate Dip-Coating of Vanadium Oxide Thin Films

Colm Glynn[1,2], Donal Creedon[1], Hugh Geaney[1,2], Eileen Armstrong[1,2], Timothy Collins[1], Michael A. Morris[1,3] and Colm O'Dwyer[1,2]

[1] *Department of Chemistry, University College Cork, Cork, Ireland*
[2] *Micro & Nanoelectronics Centre, Tyndall National Institute, Lee Maltings, Cork, Ireland*
[3] *Centre for Research on Adaptive Nanostructures and Nanodevices (CRANN), Trinity College Dublin, Dublin, Ireland*

Supporting Information

Section 1: Vanadium Oxide Thin Film Additional Images and Spectra

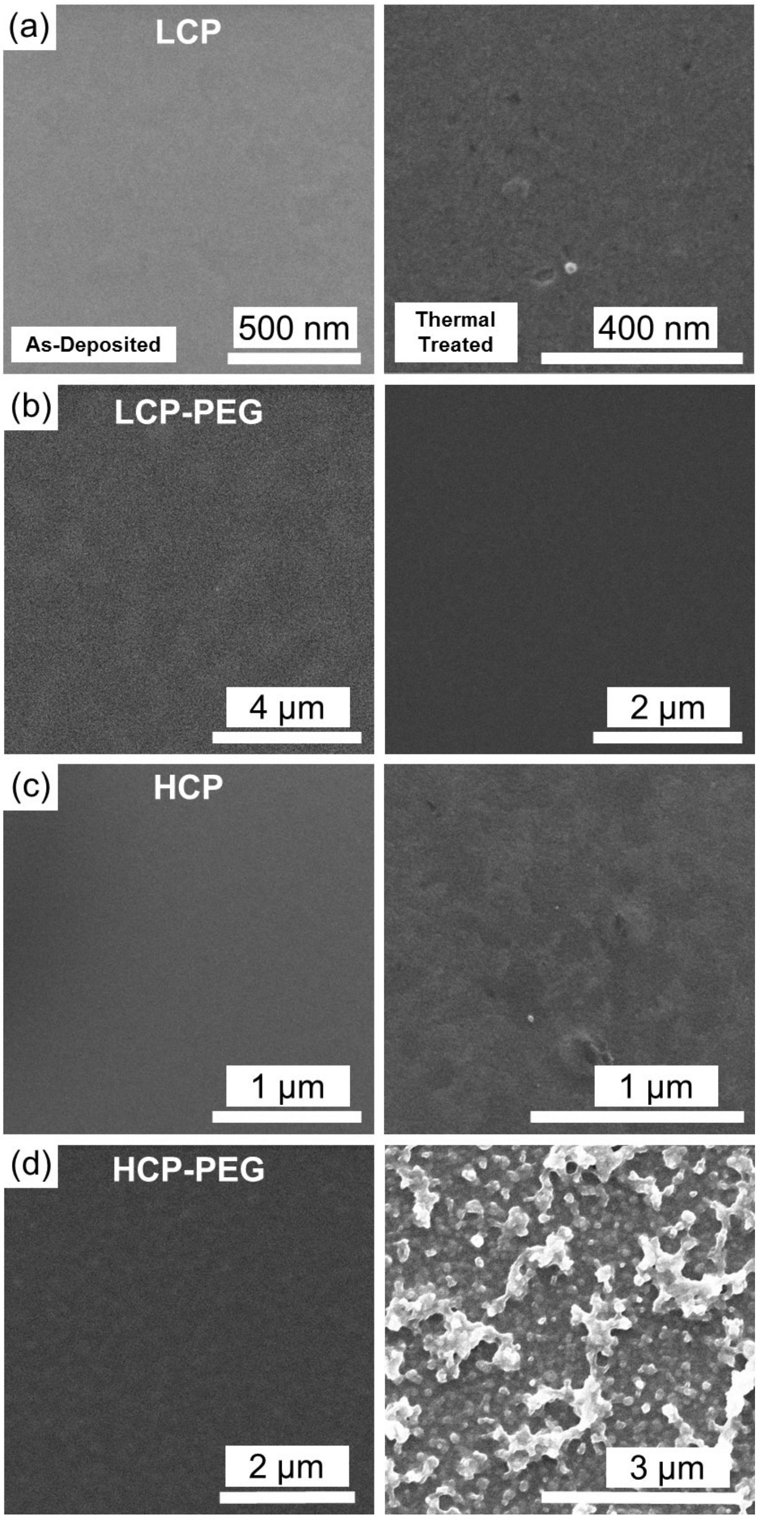

Figure S1. SEM images of the as-deposited (left) and thermally treated (right) at 300 ºC (a) LCP, (b) LCP-PEG, (c) HCP, (d) HCP-PEG thin films.

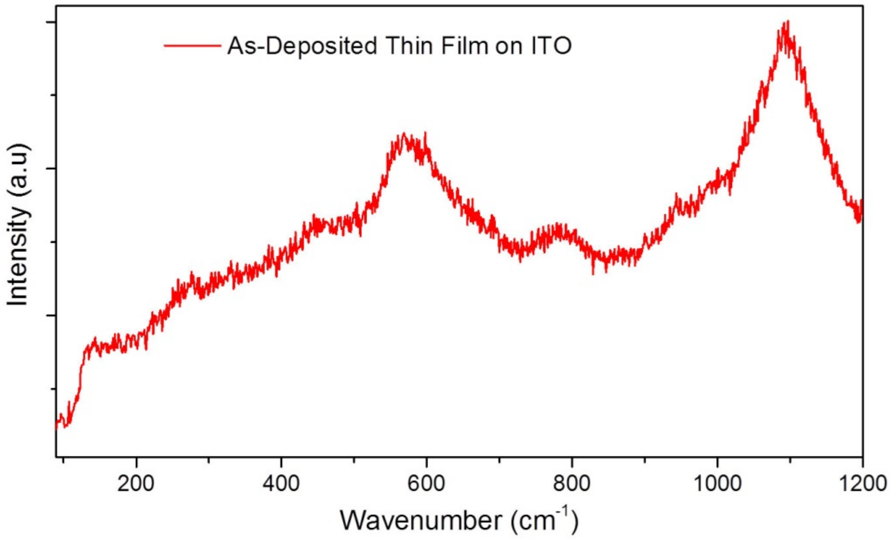

Figure S2. Raman scattering spectra of an as-deposited thin film of VO dip-coated onto an ITO substrate prior to thermal treatment.

Section 2: V_2O_5 Drop-Casted Deposits

By drop casting onto the ITO substrate, the bulk-like properties of the precursor were studied. In the drop-casting test, the LCP and LCP-PEG precursors were dropped onto the substrate and the resulting deposits were studied both pre- and post-thermal treatment. The precursor was dropped onto the substrate as a liquid and hydrolysed slowly due to the large amount of precursor present and the need for the solvent to evaporate, resulting in a slow rate of hydrolyzation. During the initial hydrolysis stage, the liquid precursor was able to flow unconstrained on the substrate prior to solidifying and forming a solid deposit. The effect of increased concentration on the drop casted samples was found to hasten hydrolysis of the deposit, the morphology of the deposit was found to be dependent instead upon the constituents of the precursor.

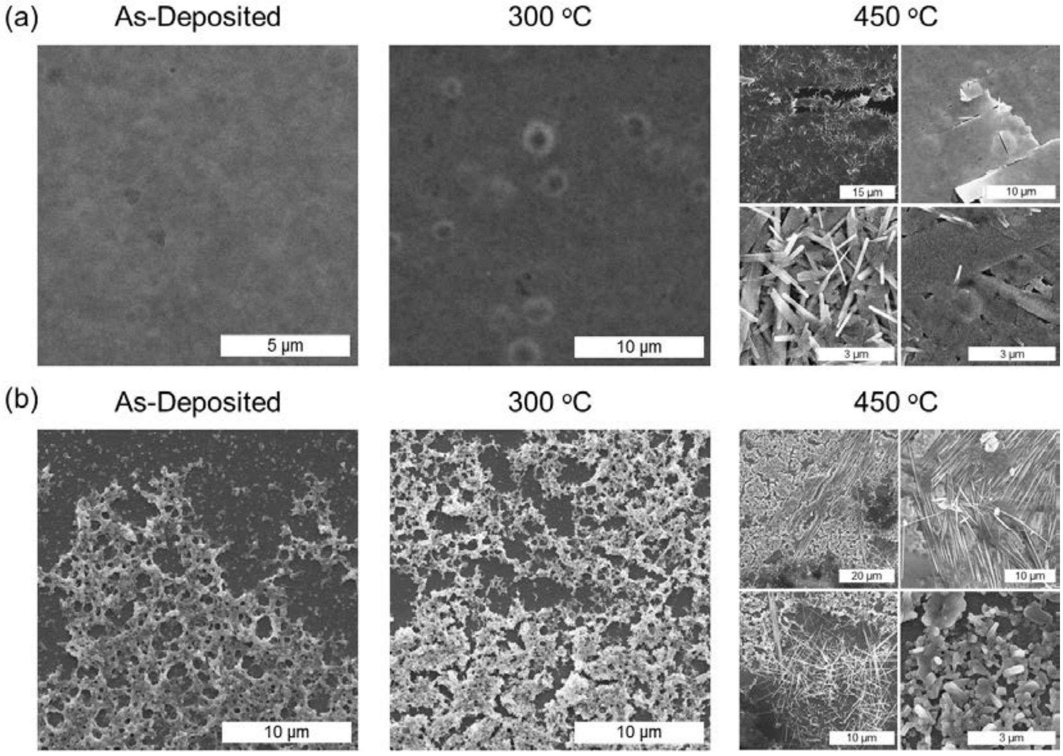

Figure S3. SEM surface images showing the surface morphology of as-deposited and thermally treated drop-casted films of VO deposits formed from (a) LCP and (b) LCP-PEG precursors. SEM images of each of the deposits formed at both a thermal treatment of 300 ^{o}C and 450 ^{o}C for 14 hours respectively, is shown.

The drop-casted deposits were thermally treated at two different temperatures, 300 ^{o}C and 450 ^{o}C, under ambient conditions. The deposits were thermally treated at 300 ^{o}C as it is

the temperature at which the thin films crystallized to orthorhombic V_2O_5 without subsequent damage to the film which was found to occur at higher temperatures, where the thin films would experience delamination and surface cracking at temperatures higher than 300 ^{o}C. A thermal treatment at the increased temperature was performed to correspond to the common crystallising temperatures from the literature.[1-2]

Surface SEM images of the as-deposited and post-thermally treated LCP and LCP-PEG samples highlighting the differences in surface morphology at both crystalizing temperatures are shown in Fig. S3 (a,b) respectively. The morphology of the as-deposited deposits is shown to be affected by the additive. The morphology of the as-deposited surfaces for the LCP and LCP-PEG samples differ. The LCP samples form a smoother surface than the LCP-PEG samples which has a rough and irregular morphology. After thermal treatment at 300 ^{o}C (the temperature used to crystallise the corresponding thin films) the morphology of the LCP samples roughens slightly with the formation of surface defects in the form of pinholes due to the densification of the sample during the thermal treatment. The morphology of the LCP-PEG samples heated at 300 ^{o}C however undergoes a larger change which results in an increased surface roughness.

After thermal treatment at 450 ^{o}C both samples show the formation of different structures of VO. The surface of the LCP samples roughens further with increased temperature and also shows the formation of rod-like structures on the surface. The same rod structures are seen in the corresponding LCP-PEG samples, however, the length of the rod structures is larger in the LCP-PEG samples. There is also the formation of crystallites on the surface of the LCP-PEG samples heated at 450 ^{o}C. The rougher morphology of the thermally treated LCP-PEG sample is attributed to the demixing and removal of the PEG during the thermal treatment as the VO crystallizes. The PEG demixes from the as-deposited stoichiometric deposit as the temperature increases and is then removed as the temperature further increases. During the removal process, the surface of the crystallising V_2O_5 fractures, resulting in the surface morphology seen in Fig. S3 (b). Crystalline rods are a common morphology for orthorhombic V_2O_5,[3] however, the size variations of the rods and other crystallites seen on the surface may be indicative of the presence of other forms of VO which are to be studied using XRD and Raman scattering spectroscopy.

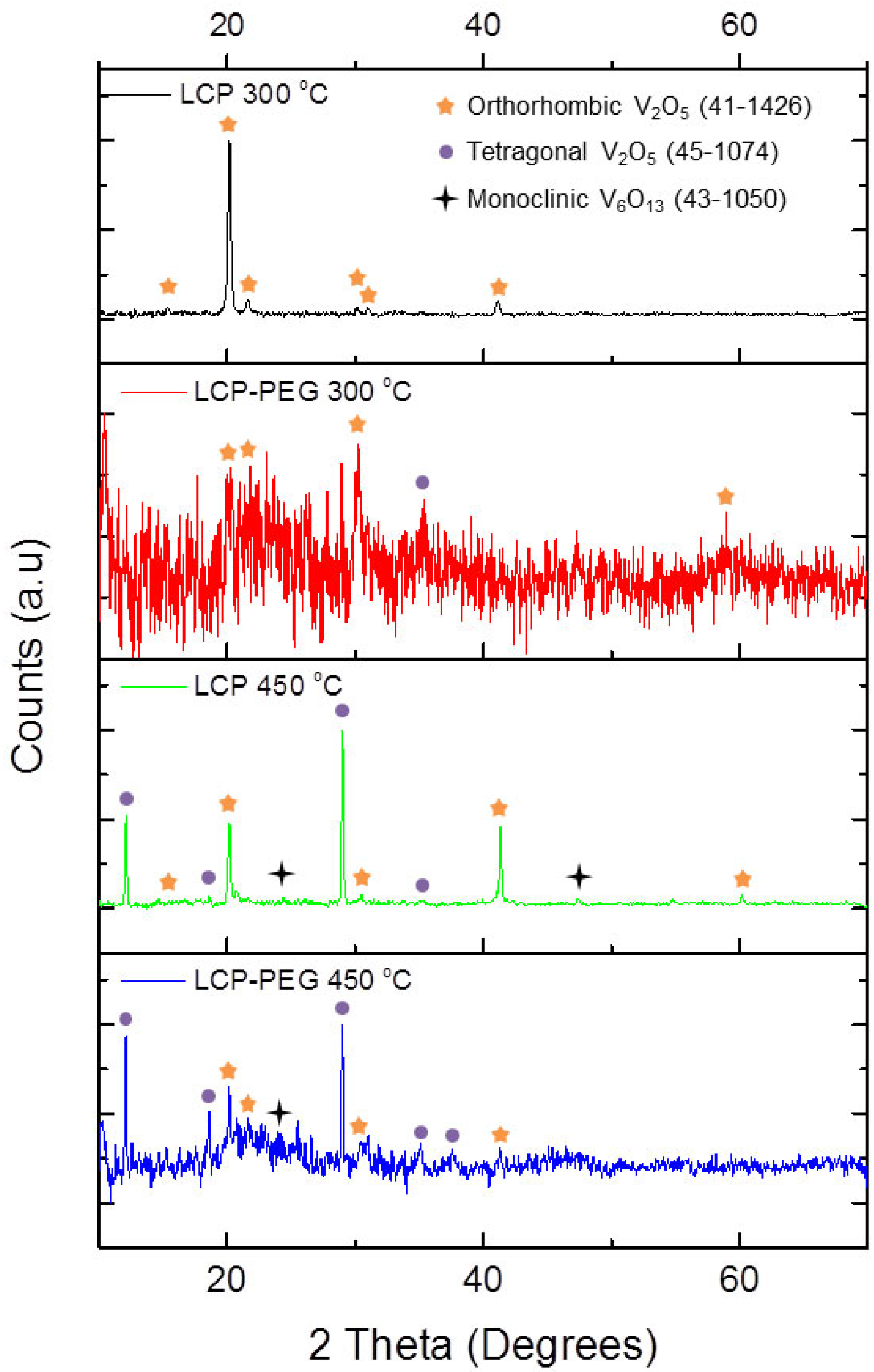

Figure S4. XRD patterns of LCP and LCP-PEG drop-casted films thermally treated at 300 °C and 450 °C for 14 hours. The indexed XRD patterns for a variety of VO oxidative states are highlighted.

Figure S4 shows the XRD patterns of the drop-casted deposits after thermal treatment. XRD analysis of the deposits after thermal treatment at 300 °C shows the formation of predominantly orthorhombic V_2O_5 for the LCP deposit with small amounts of tetragonal V_2O_5 also found in the LCP-PEG deposit. After thermal treatment at 450 °C both the LCP

and LCP-PEG deposits show the formation of orthorhombic and tetragonal V_2O_5 with small amounts of V_6O_{13} also apparent in the deposits.

The Raman spectra for the LCP and LCP-PEG deposits treated at 300 °C are shown in Fig. S5 (a). The Raman spectra show the formation of orthorhombic V_2O_5. The Raman scattering spectra for two regions of the LCP deposits formed at 450 °C are presented in Fig. S5 (b) and show the formation of predominantly orthorhombic V_2O_5 with some areas exhibiting some deformation to the highlighted regions due to the presence of some other vibrations due to different VO phases, in this case most probably that of tetragonal V_2O_5.

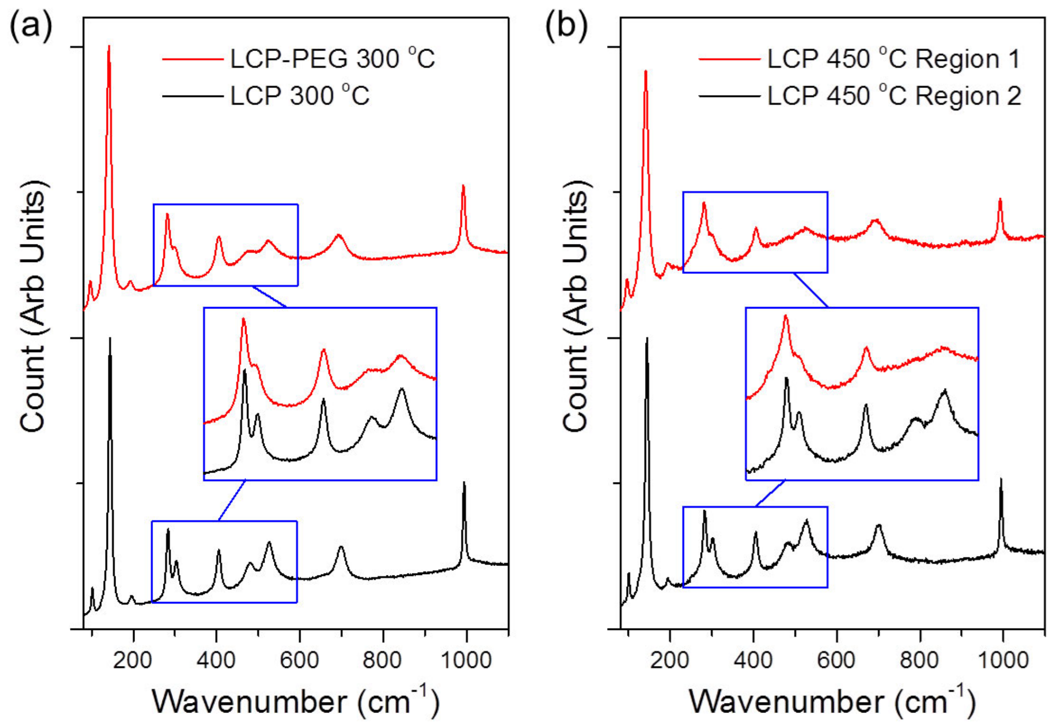

Figure S5. Raman scattering spectroscopy of drop casted deposits of (a) LCP and LCP-PEG thermally treated at 300 °C showing the formation of V_2O_5. (b) Two regions from the LCP deposit thermally treated at 450 °C showing the deformation of regions the V_2O_5 vibrations due to the presence of small quantities of different VO phases.

As seen in the SEM images in Fig. S3, the LCP-PEG deposits underwent a large surface change in different regions of the deposit during thermal treatment at 450 °C with the formation of long crystalline rods and crystallites. The Raman scattering spectra for three regions on the surface of the deposit is presented in Fig. S6 (a). The Raman spectra for region one is that of orthorhombic V_2O_5 and is the dominant spectrum found in the deposit, however, areas such as region two and three are interspersed through the surface and the vibrations differ from those of orthorhombic V_2O_5. Figure S6 (b) is a deconvolution of the

Raman scattering spectrum for region two and three. The corresponding vibrations which make up the spectra were extracted and used to help determine the VO phases which are present. The vibrations from the deconvoluted spectra were not correlated to one VO phases in particular, the vibrations of both region two and three were instead attributed to the vibrations from a combination of V_6O_{13}, V_2O_3 and VO_2 respectively.[4-5]

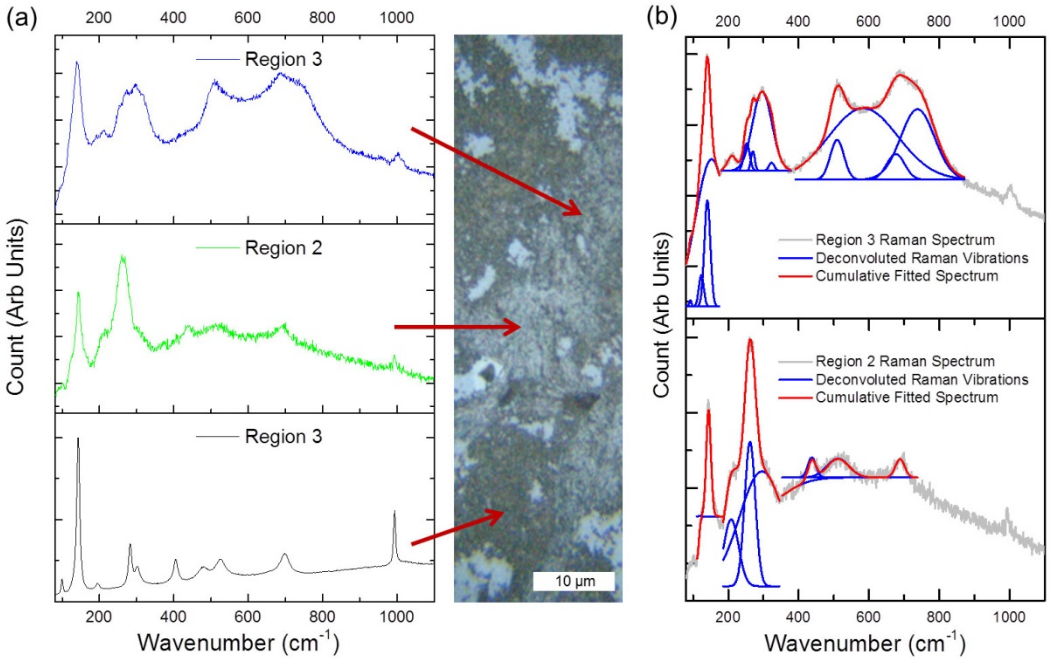

Figure S6. (a) Raman scattering spectra and image of the different regions of the LCP-PEG samples thermally treated at 450 °C showing the varied spectra found at each region. (b) Deconvolution of the Raman scattering spectra for region 2 and 3 to characterize the contributing vibrations of the spectra.

The presence of other phases of VO in the XRD spectra is attributed to the crystallization of the disordered as-deposited material. The disorder in the material and likely facilitate crystallization at different rates forming small amounts of different phases of VO. The lack of evidence for different phases of VO in the Raman scattering spectra shows that the amounts of the other VO phases in the deposit are minimal and are more easily detected in a larger area by XRD.

Morphological and structural analysis of the drop-casted V_2O_5 samples provided information on the formation of other phases within the samples at different temperatures. At the lower 300 °C crystallising temperature, the drop casted samples showed that traces of other VO phases can form in the samples; this was applicable to the corresponding thin films for which the smaller amount of other VO phases proved to be difficult to quantify.

Section 3: In-Plane TEM Images

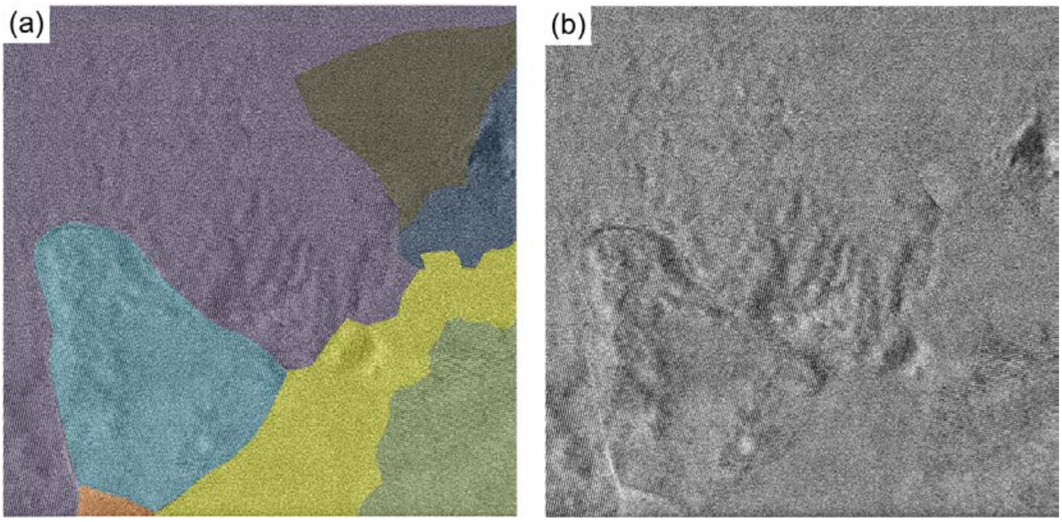

Figure S7. (a) TEM image of the different crystalline grains of the LCP thin films thermally treated at 300 °C on a Si_3N_4 coated TEM grid (b) corresponding TEM image without the grains highlighted.

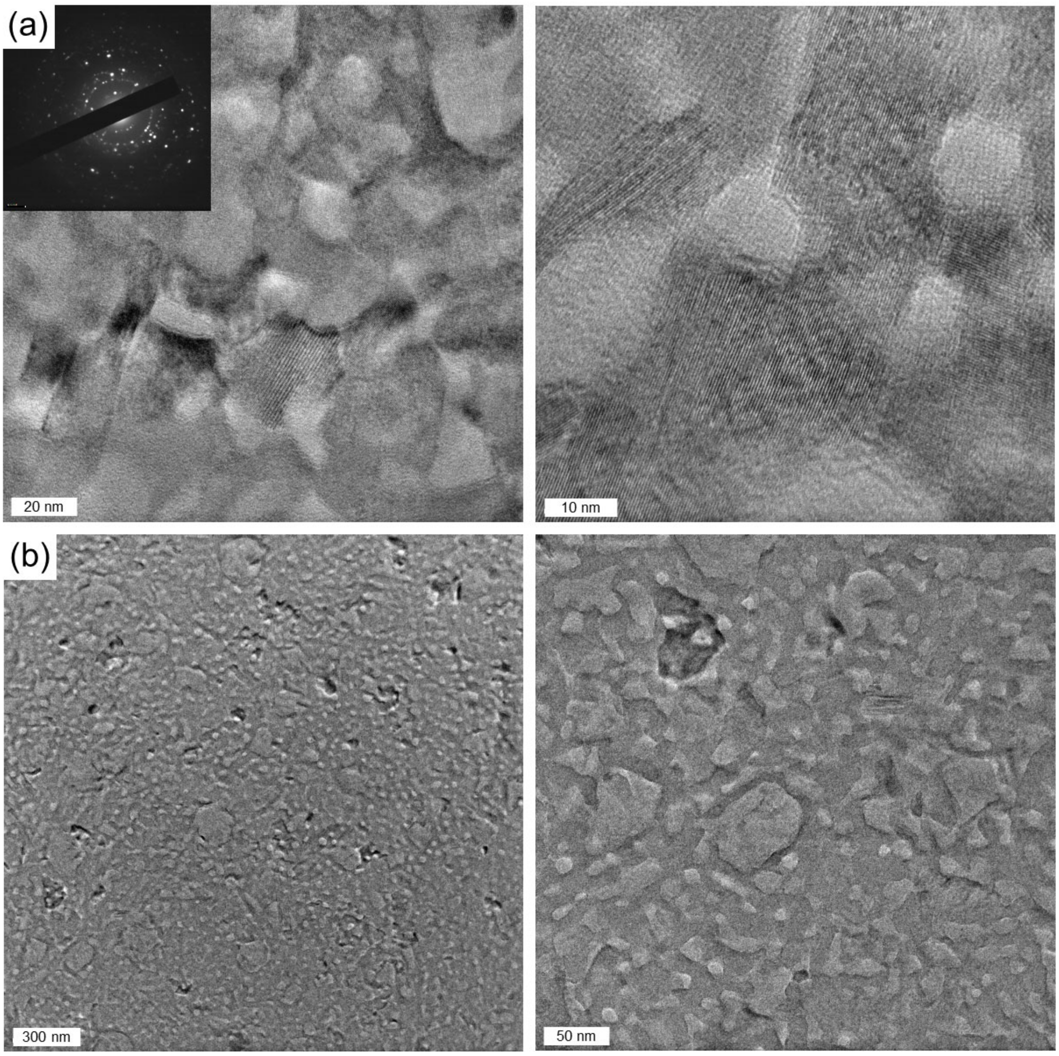

Figure S8. TEM images of different regions of the LCP-PEG thin film deposited onto Si_3N_4 TEM grids. These regions show the formation of porous thin films of the V_2O_5 attributed to the effects of the PEG on the crystallization of the thin film.

Section 4: Thin Film Model Calculation

Fast and Slow-Rate Thin Film Deposition

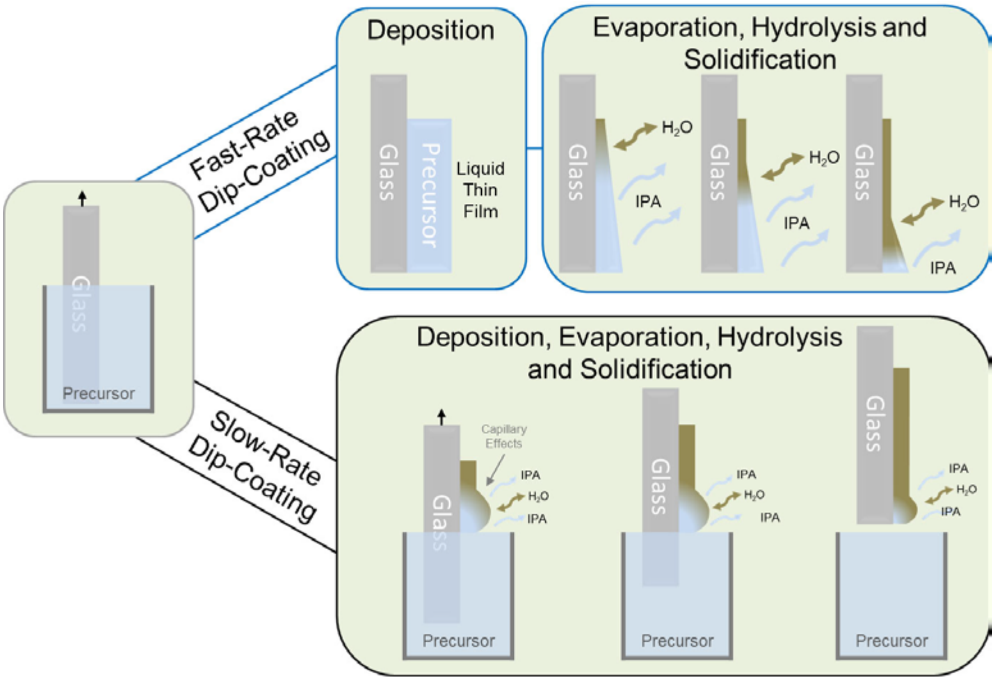

Figure S9. Formation processes of fast- and slow-rate dip-coated thin films.

The formation process for thin films deposited using fast- and slow-rate dip-coating are shown in Fig. S9. At fast-rates, the liquid film is deposited on the surface of the substrate prior to the evaporation, hydrolysis and subsequent formation of the solid thin film. At slow-rates for dip-coating, the same evaporation, hydrolysis and solidification processes occur whilst the substrate is still being withdrawn from the precursor. Capillary action effects the formation of the thin film as it is still in contact with the meniscus of the liquid.

Deposited Liquid Thin Film

In the fast-rate dip-coating regime used in this work, the Landau-Levich equation is applicable for estimating the deposited liquid thin film thickness.[6-7]

h = liquid film thickness

η = viscosity

u = dip-coating speed

g = standard gravity

γ = surface tension

ρ = density of fluid

$$h = \frac{0.94\,\eta^{2/3}}{\gamma^{1/6}\,(\rho g)^{1/2}}\,u^{2/3}$$

From this equation the thickness of a liquid thin film on the substrate can be estimated. In this work, a thin film of a precursor composed of IPA, V_2O_5 alkoxide and an additive of either H_2O or PEG-400 is dip-coated onto a substrate. Two IPA/Alkoxide/Additive concentrations were tested, a low and high concentration where the mixture mixed by volume was 1000:10:1 and 250:10:1 respectively.

At both concentrations the impact of the alkoxide and additive on the wetting characteristics of the IPA is negligible due to their small comparative volume. Therefore, the thickness of the IPA film at a withdrawal speed of 2.5 mm/s can be calculated by using the constants for IPA by itself. When this is done the thickness of the initially deposited liquid film on the surface of the substrate can be estimated.

η = 2.3703 mPa.s = 2.3703×10^{-3} kg/ms

γ = 0.0217 N/m = 2.212×10^{-3} kg/m

u = 2.5 mm/s = 2.5×10^{-3} m/s

ρ = 0.786 g/ml = 786 kg/m^3

g = 9.8 m/s^2

Calculated IPA liquid film thickness, h = 9.7163×10^{-6} = 9.7163 µm

<u>Material proportion constant (k)</u>

The k value is related to the amount of the resulting solid thin film that is within the liquid precursor and is different for each precursor. The k value can be calculated (k_C) through the concentration and density of the solid material within the film.[7-8] It can also be calculated by using the experimental data for thickness for each precursor (k_E)

$$k_C = \frac{C_i\ M_i}{\alpha_i\ \rho_i}$$

C_i = inorganic precursor concentration (mol/cm^3)

α_i = fraction of material in the film (%)

M_i = inorganic material molar weight (g/mol)

ρ_i = density of the inorganic material (g/cm^3)

(Note: α_i was originally calculated by Faustini *et. al.* through comparison of the initial and final refractive index of the material.[8] As the value is given as "the fraction of material in the

liquid film", the percentage amount of the vanadium oxide and additive was calculated through their respective molar masses within each precursor and used in calculating the k_C value)

Table S1. Calculated *k* values for each of the precursors:

**Precursor**	**k_C**	**k_E**	**k_C / k_E**
LCP	0.76356	1.6858×10^{-3}	452.936
LCP-PEG	0.75494	3.5248×10^{-3}	214.170
HCP	0.79618	4.0756×10^{-3}	195.353
HCP-PEG	0.76275	7.1323×10^{-3}	106.943

The k_E value is two orders of magnitude smaller than k_C. It can therefore be assumed that the solid thin film which results from the initial liquid thin film cannot be calculated through a simple relationship between the "solid" materials in the precursor; instead, the thin film forms through the evaporation and phase change that occurs after deposition during this time. The experimentally calculated k_E value incorporates this process for each precursor.

References

1. Goltvyanskyi, Y. *et al.* Structural transformation and functional properties of vanadium oxide films after low-temperature annealing. *Thin Solid Films* ***564***, 179-185 (2014).
2. Li, Z. *et al.* Ir Detectors: Ultrahigh Infrared Photoresponse from Core–Shell Single-Domain-VO_2/V_2O_5 Heterostructure in Nanobeam *Adv. Func. Mater.* ***24***, 1820-1820 (2014).
3. Pelletier, O. *et al.* A Detailed Study of the Synthesis of Aqueous Vanadium Pentoxide Nematic Gels. *Langmuir* ***16***, 5295-5303 (2000).
4. Kuroda, N.; Fan, H. Y. Raman scattering and phase transitions of V_2O_3. *Phys. Rev. B* ***16***, 5003-5008 (1977).
5. Chen, X.-B.; Shin, J.-H.; Kim, H.-T.; Lim, Y.-S. Raman analyses of co-phasing and hysteresis behaviors in V2O3 thin film. *J. Raman Spectrosc.* ***43***, 2025-2028 (2012).
6. Landau, L.; Levich, B. Dragging of a liquid by a moving plate. *Acta Physicochim. URSS* ***17***, 12 (1942).
7. Faustini, M. *et al.* Preparation of Sol−Gel Films by Dip-Coating in Extreme Conditions. *J. Phys. Chem. C* ***114***, 7637-7645 (2010).
8. Faustini, M. *et al.* Engineering Functionality Gradients by Dip Coating Process in Acceleration Mode. *ACS Appl. Mater. Interfaces* ***6***, 17102-17110 (2014).
